# KR-12-a5 Reverses Adverse Effects of Lipopolysaccharides on HBMSC Osteogenic Differentiation by Influencing BMP/Smad and P38 MAPK Signaling Pathways

**DOI:** 10.3389/fphar.2019.00639

**Published:** 2019-06-05

**Authors:** Hui Li, Shutao Zhang, Bin’en Nie, Teng Long, Xinhua Qu, Bing Yue

**Affiliations:** Department of Bone and Joint Surgery, Renji Hospital, Shanghai Jiaotong University School of Medicine, Shanghai, China

**Keywords:** KR-12-a5, LPS (lipopolysaccharide), osteogenic differentiation, p38 mitogen-activated protein kinase, BMP/Smad signal

## Abstract

KR-12-a5 is an analogue of the antimicrobial peptide KR-12. Both of these two agents can play key effects in the treatment of infections such as osteomyelitis. Our previous work demonstrated that the osteogenic differentiation of human bone marrow mesenchymal stem cells (HBMSCs) can be enhanced by KR-12. The present study investigated if KR-12-a5 could reverse the adverse effects of lipopolysaccharides (LPS) on HBMSC osteogenesis and the involved molecular mechanisms. We observed the proliferation, cell cycle, and apoptosis of HBMSCs in the presence of KR-12-a5 by a cell counting kit-8 assay and flow cytometry. The osteogenic differentiation of HBMSCs was studied by alkaline phosphatase, Alizarin Red staining, and quantitative assays. Osteogenic differentiation marker levels were detected using real-time quantitative PCR analysis, which demonstrated that KR-12-a5 treatment reversed the inhibition of osteogenesis. Western blot analysis indicated that LPS-activated P38 mitogen-activated protein kinase (MAPK) signaling was inhibited and BMP/Smad pathway was reactivated after KR-12-a5 treatment under induced osteogenic conditions. Furthermore, flow cytometry results demonstrated that KR-12-a5 relieved LPS-induced oxidative stress. Combining the LPS-treated mouse model results, we proved that KR-12-a5 reversed the adverse effects of LPS on HBMSC osteogenic differentiation by influencing the BMP/Smad and P38 MAPK signaling pathways.

## Introduction

Local or systemic complications can be caused by infectious diseases such as osteomyelitis. Gram-negative bacteria are common in inflammatory bone diseases such as chronic osteomyelitis and tend to be protracted and persistent (Lesse et al., 1987; Carvalho et al., 2012). Lipopolysaccharides (LPSs) are components of the gram-negative bacterial cell membrane and are considered to be important factors in chronic inflammatory processes. They are reported to induce osteolysis *in vitro* and *in vivo* (Orcel et al., 1993; Chiang et al., 1999; Itoh et al., 2003; Islam et al., 2007; Mormann et al., 2008). Furthermore, LPS also inhibits human bone marrow mesenchymal stem cell (HBMSC) and osteoblast osteogenic differentiation, which makes it difficult to recover bone loss (Kadono et al., 1999; Bandow et al., 2010; Xing et al., 2010). The LPS-induced local inflammatory environment leads to an increase in a series of inflammatory factors and local oxidative stress levels, which is not conducive to the formation of a localized osteogenic microenvironment (Guo et al., 2014, 2015; Wang et al., 2017). Systemic or topical administration of antibiotics is commonly used to clinically treat osteomyelitis and gram-negative bacterial infections (Spellberg and Lipsky, 2012; Bernard et al., 2015). However, antibiotics commonly used in the treatment of osteomyelitis have resulted in an increased emergence of bacterial resistance and are difficult to solve the osteolysis caused by LPS and other inflammatory factors (Le Clerc et al., 2014). Therefore, the discovery of good antibacterial agents with the ability to reverse inflammatory environments and promote new bone formation will play a key role in clinical treatment.

The natural antibacterial peptide in the human body, cathelicidin, attracted great attention due to its effects to physically destroy bacterial membranes and cause dissolution, and was considered a promising alternative to traditional antibiotics (Boman, 1995; Zasloff, 2002). The C-terminal antimicrobial region of human cathelicidin (LL-37; LLGDFFRKSKEKIGKEFKRIVQRIKDFLRNLVPRTES) plays an important role in the response to local and systemic pathogen invasion (Frohm et al., 1997; Dorschner et al., 2001; Ramos et al., 2011). In monocyte-like or macrophage-like cell lines, the production of diverse pro-inflammatory cytokines including interleukin 1β (IL-1β), tumor necrosis factor α (TNF-α), interleukin 6 (IL-6), and nitric oxide (NO) was apparently inhibited by LL-37 in response to LPS (Scott et al., 2000; Nagaoka et al., 2002). Previous studies showed that LL-37 suppressed 106 out of the 125 LPS-upregulated genes in human monocytes (Bucki et al., 2010). Moreover, it also promotes the chemokine activation of peripheral blood-derived monocytes by activating P38 (P38 kinase) and extracellular signal-regulated kinase 1/2 (ERK1/2) (Tjabringa et al., 2003; Bowdish et al., 2004). However, due to its long amino acid sequence, LL-37 is not suitable as a conventional treatment for infectious and inflammatory diseases. (Jacob et al., 2013). Compared with LL-37, AMPs with short sequence attract more attention because they have showed less toxic effects on eukaryotic cells and less interaction with human plasma proteins. What’s more, their production costs are lower (Ciornei et al., 2005). Two LL-37 derivatives (KR-20; KRIVQRIKDFLRNLVPRTES; residues 18–37 of LL-37 and KS-30; KSKEKIGKEFKRIVQRIKDFLRNLVPRTES; residues 8–37 of LL-​37) exhibit increased antimicrobial activities when compared to native LL-37 (Murakami et al., 2004). As the shortest peptide that demonstrates antibacterial activity and retains the core amphipathic helical structure of LL-37, KR-12 (KRIVQRIKDFLR; residues 18–29 of LL-37) has the advantages of low synthesis cost and low cytotoxicity (Sigurdardottir et al., 2006; Wang, 2008; Mishra et al., 2013). Moreover, KR-12 does not lyse human red blood cells unlike LL-37, which produces a certain hemolytic effect (Jacob et al., 2013; Mishra et al., 2013). Our previous studies demonstrated KR-12 to promote HBMSC osteogenic differentiation while possessing good antibacterial properties (Li et al., 2018). As an analogue of KR-12, KR-12-a5 (KRIVKLILKWLR) has been reported to exhibit better antibacterial properties against clinically resistant bacteria while maintaining good biocompatibility. It also reduces inflammation and inhibits the secretion of inflammatory factors (Kim et al., 2017). Therefore, KR-12-a5 is expected to become a good mode of treatment for gram-negative bacteria-induced osteomyelitis and LPS-induced osteolysis.

HBMSCs play an important role in the renewal of osseous tissue and are the prime source of osteoprogenitor cells. The bone-forming osteoblasts can be differentiated from HBMSCs (Bielby et al., 2004; Karner et al., 2009). After the local pathogens are cleared, HBMSCs are activated to differentiate into osteoblasts to repair local osteolysis. If HBMSCs are inhibited by the inflammatory environment, local bone defects will be difficult to repair and pathological fractures may occur (Wagner and Hansch, 2015). In the osteoblast-differentiating process, HBMSCs are regulated by a variety of signaling molecules and pathways that allow them to exhibit various differentiation behaviors in different microenvironments. As a member of the transforming growth factor (TGF-β) superfamily, bone morphogenetic proteins (BMPs) are potent factors of osteoblastic differentiation in osseous formation (Mukherjee et al., 2010; Rider and Mulloy, 2010). After the binding of BMP and transmembrane receptors, intracellular Smad proteins are the major members of signal transduction. Activated Smad 1/5/9 is further binging with Smad 4, transporting to the nucleus and activating transcription of osteogenic related genes (Yamazaki et al., 2009). Several mitogen-activated protein kinases (MAPKs), such as P38 and ERK1/2, have also been reported to possess the function of regulating the differentiation of HBMSCs (Zhang and Liu, 2002; Salasznyk et al., 2004). Furthermore, ERK1/2 and P38 MAPK signaling also activate the transcriptional activity of Runt-related transcription factor 2 (Runx2) (Xiao et al., 2002; Franceschi and Xiao, 2003). As signaling molecules that are activated under inflammatory conditions, MAPKs are often activated by pro-inflammatory substances such as LPS (Yeom et al., 2015). When localized osteogenic microenvironments are affected by inflammatory reactions, MAPKs, Akt/mTOR/4EBP1, P53, and NF-κB signal transductions are also involved in the inflammation-induced inhibition of osteogenic differentiation (Martindale and Holbrook, 2002; Zeng et al., 2014).

Since KR-12 and several other antibacterial peptides have the ability to promote stem cell differentiation (Krasnodembskaya et al., 2010; Alcayaga-Miranda et al., 2017; Milhan et al., 2017; Li et al., 2018), KR-12-a5 may also have the potential to promote HBMSC osteogenic differentiation. Associated with the excellent anti-inflammatory properties of this small peptide, KR-12-a5-mediated regulation of osteogenesis can be expected under inflammatory conditions. In this study, KR-12-a5 was synthesized and its effect on osteogenic differentiation in LPS-induced HBMSCs was investigated *in vitro* and *in vivo*. Given the importance of BMP/Smad and MAPK signaling in osteogenic differentiation and inflammation processes, the molecular mechanisms involved in KR-12-a5 treatment were also explored.

## Materials and Methods

### Cell Culture and Identification

All experiments were performed in accordance with the guidelines and approved by the ethics committee at Renji Hospital, Shanghai Jiaotong University School of Medicine. The registration number of the ethics committee is SHNHEA[2015]75. Informed consents were obtained from human participants. HBMSCs were isolated and expanded as previously described (Tang et al., 2002). Six-milliliter bone marrow aspirates were harvested in heparin (about 200 U/ml total) from the iliac crest of a healthy adult donor. Then the bone marrow was diluted in α-MEM (Hyclone, Logan City, Utah, USA) containing 10% (v/v) FBS (Gibco, Carlsbad, CA, USA). The suspension was seeded into T-25 cell culture flasks at 3 ml of bone marrow per flask.

Several monoclonal antibodies including PE-CyTM7-CD90, APC-CD105, PE-CD34, and PE-CD45 (BD Biosciences, San Diego, CA, USA) were used for cell phenotyping identification through flow cytometry. Non-specific staining was distinguished by isotype controls. HBMSCs were incubated with antibodies for 30 min. After two times washing process, the cell suspensions were analyzed by flow cytometer (FACSCalibur^™^, BD Biosciences, Franklin Lakes, NJ, USA).

### Analysis of Cytotoxicity, Cell Cycle, and Apoptosis

The cytotoxicity of KR-12-a5 (GL Biochemistry, Shanghai, China) was assessed by a CCK-8 test (Donjindo, Japan). Briefly, various concentrations of KR-12-a5 (0, 1, 2, 4, 8, 16, 32, or 64 μg/ml) were added into 96-well plates plated with HBMSCs (5.0 × 10^3^ cells/well) for 1, 3, or 5 days. At each time point, the cell culture medium was removed and 100 µl of 10% CCK-8 solution was added to each well. The absorbance of the solution was tested at 450 nm after 2 h.

PI/RNase staining (BD Biosciences, San Diego, CA, USA) was used to test the cell cycle of HBMSCs after KR-12-a5 incubation. Briefly, after 48 h of KR-12-a5 treatment, HBMSCs were fixed with 75% ethanol at −20°C for 2 h. After fixation, the cells were resuspended in 0.5 ml of PI/RNase staining buffer and incubated for 15 min. The cell cycle proportions (G0, G1, S, and G2-M phases) were tested by a flow cytometer.

Apoptosis was assessed using flow cytometry while double-staining for Annexin V-FITC and PI (BD Biosciences, San Diego, CA, USA). HBMSCs were collected after 48 h of KR-12-a5 treatment and stained with 200 μl of 1× binding buffer containing 5 μl of Annexin V-FITC and 10 μl of PI for 15 min.

### The LPS-Agent Culture System

Related experiments with pre-LPS stimulation were carried out using the LPS-agent culture system. After cell attachment, HBMSCs were incubated with serum-free medium or LPS (1 μg/ml, Sigma, Louis, MO, USA) for 24 h to induce inflammatory responses. After washing with PBS, various concentrations of KR-12-a5 solution using osteogenic induction medium as a solvent were added to the plate (Wang et al., 2016). HBMSCs pretreated with LPS or LPS/KR-12-a5 served as the treatment groups, while HBMSCs pretreated with serum-free medium were used as the negative control groups.

### Assessment of ALP Activity and Mineralized Matrix Formation

HBMSCs (5 × 10^4^ cells/well) were seeded into 24-well plates. After 7 days KR-12-a5 treatment, HBMSCs were subjected to ALP staining. Cells were fixed for 30 s and stained with the ALP staining kit (Sigma, Louis, MO, USA) for 15 min. Quantitative analysis of ALP in HBMSCs was done by ALP activity kit (Beyotime Biotechnology, Nantong, China). Cells were lysed with Triton X-100 (1%) and ALP activity was tested at 405-nm absorbance. Additionally, the mineralized matrix deposition in HBMSCs was assessed by Alizarin Red staining (Servicebio, Wuhan, China) after 21 days of KR-12-a5 treatment. Cells were fixed with 4% paraformaldehyde for 15 min, and then 500 μl Alizarin Red Dye Solution was added into 24-well plates for 30 min. A 10% chlorinated 16 alkyl pyridine solution of sodium phosphate (pH 7.0, Sigma, Louis, MO, USA) was used as a dissolving solution for quantitative analysis. The absorbance of the stained eluent was detected at 620 nm.

### Quantitative Reverse Transcription PCR (RT-qPCR)

After 3, 7, and 14 days of KR-12-a5 treatment, TRIzol reagent (Life Technologies, Carlsbad, CA, USA) was used for the extraction of total RNA in HBMSCs. PrimeScript^™^ RT Master Mix (TaKaRa, Japan) was used for cDNA synthesis. Real-time PCR was performed using SYBR^®^ Premix EX Taq^™^ (TaKaRa, Japan). The primer sequences used in the experiment are as follows: GAPDH, 5’-GGAGCGAGATCCCTCCAAAAT-3’ (forward) and 5’-GGCTGTTGTCATACTTCTCATGG-3’ (reverse); RUNX2, 5’-TGGTTACTGTCATGGCGGGTA-3’ (forward) and 5’-TCTCAGATCGTTGAACCTTGCTA-3’ (reverse); ALP, 5’-ACCACCACGAGAGTGAACCA-3’ (forward) and 5’-CGTTGTCTGAGTACCAGTCCC-3’ (reverse); COL1A1, 5’-GAGGGCCAAGACGAAGACATC-3’ (forward) and 5’-CAGATCACGTCATCGCACAAC-3’ (reverse); BSP, 5’-CACTGGAGCCAATGCAGAAGA-3’ (forward) and 5’-TGGTGGGGTTGTAGGTTCAAA-3’ (reverse); OCN, 5’-CACTCCTCGCCCTATTGGC-3’ (forward) and 5’-CCCTCCTGCTTGGACACAAAG-3’ (reverse); OPN, 5’-CTCCATTGACTCGAACGACTC-3’ (forward) and 5’-CAGGTCTGCGAAACTTCTTAGAT-3’ (reverse); BMP2, 5’-GGTATCACGCCTTTTACTGCC-3’ (forward) and 5’-ACACCCACAACCCTCCACAA-3’ (reverse); and OSX, 5’-ACACTGGGCAGACAGTCAG-3’ (forward) and 5’-CCCTTTACAAGCACTAATGG-3’ (reverse).

### Western Blot Analysis

RIPA lysis buffer containing 1× PMSF (Beyotime Biotechnology, Nantong, China) and 1× protease inhibitor cocktail (Life Technologies, Carlsbad, CA, USA) was used as a lysis reagent for HBMSCs in six-well plates. After shaking on ice and centrifuging, the concentration of total protein was determined by BCA assay. The proteins in each group were separated using 10% sodium dodecyl sulfate (SDS)–polyacrylamide gels (EpiZyme, Shanghai, China) and transferred to polyvinylidene fluoride membranes (Millipore, Billerica, MA, USA). After incubating in skimmed milk (BD Biosciences, San Diego, CA, USA) to block nonspecific binding, the membranes were further incubated with primary antibodies for 8 h and HRP-conjugated secondary antibodies for 1 h. The immunoreactive bands were visualized using the Immobilon^™^ Western HRP (Millipore, Billerica, MA, USA).

### Assessment of Reactive Oxygen Species and Superoxide Dismutase in HBMSCs

The reactive oxygen species (ROS) levels in HBMSCs induced by LPS were measured using DCFH-DA as a fluorescent probe. After 48 h of KR-12-a5 treatment, HBMSCs were incubated with DCFH-DA (10 Mm, Sigma, Louis, MO, USA) for 30 min at 37°C. Flow cytometry was used for the detection of the intensity of 2’,7’-dichlorofluorescin (DCF) fluorescence (Wang et al., 2017). After culturing with KR-12-a5 for 7 days, superoxide dismutase (SOD) activity in HBMSCs was determined by the Total Superoxide Dismutase Assay Kit (Beyotime Biotechnology, Nantong, China) and normalized to total protein concentration.

### LPS-Induced Bone Erosion

Five-week-old C57BL/6 mice were divided into three groups of five mice. LPS (5 μg/g of body weight) was injected intraperitoneally on days 0 and 4 (Wu et al., 2018). One day after LPS injection and subsequently every alternate day for up to 7 days, intraperitoneal injections of KR-12-a5 (2 μg/g of body weight) or PBS were administered. On the ninth day after the first LPS injection, the mice were sacrificed. The femur was removed and a high-resolution micro-CT analysis was performed (μCT-100) (Kim et al., 2015). The bone volume/tissue volume (BV/TV), trabecular number (Tb.N), trabecular separation (Tb.Sp), and connectivity density (Conn.D) were tested to assess the microstructure of trabecular bone using the Evaluation V6.5-3 (SCANCO Medical AG). For histological analysis, the femurs were fixed with 4% paraformaldehyde and further decalcified with 12% EDTA. Then the femurs were embedded in paraffin and sectioned. Sections were stained with H&E, Masson, and Trap. The quantitative analysis including trabecular area percentage (Tb.Ar%), Tb.Sp, collagen fiber area percentage (Col. Ar%), and number of Trap staining positive cells per unit area (NO. Trap+ Cell) were accomplished by Image-pro plus 6.0 (Media Cybernetics, Inc., Rockville, MD, USA).

### Statistical Analysis

The data are expressed as the mean ± standard deviation from at least three independent experiments. The results were analyzed *via* Student’s t-test or one-way analysis of variance using the SPSS 23.0 software (SPSS Inc., Chicago, IL, USA). P < 0.05 indicated a significant difference between groups.

## Results

### Identification of HBMSCs

Flow cytometric analysis was used to characterize the surface molecules of HBMSCs. The results showed that HBMSCs had the characteristic patterns of mesenchymal surface markers, including CD105 and CD90, and negatively expressed the hematopoietic markers, CD45 and CD34 (**Supplementary Figure 1**).

### Effects of KR-12-a5 on HBMSC Proliferation, Cell Cycle Progression, and Apoptosis

**Figure 1:**
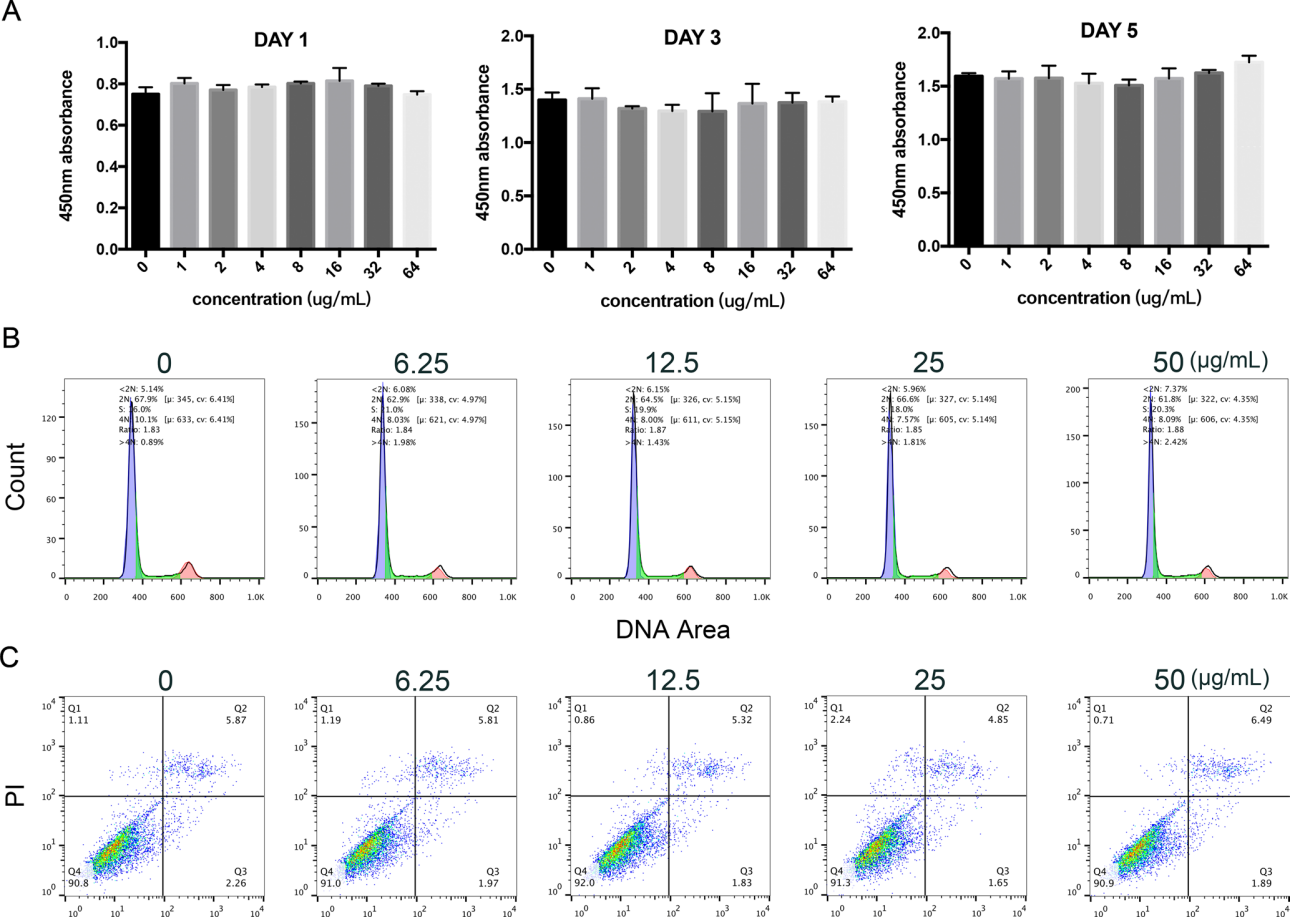
The effects of KR-12-a5 on viability of HBMSCs. **(A)** HBMSCs were treated with different concentrations of KR-12-a5 for 1, 3, and 5 days prior to measuring the cell viability by a CCK-8 test. **(B, C)** Cell cycle and cell apoptosis were carried out by flow cytometry after 48-h incubation with different concentrations of KR-12-a5.

CCK-8 analysis indicated the effect of KR-12-a5 on cell proliferation. As shown in **Figure 1A**, KR-12-a5 showed no cytotoxicity at concentrations up to 64 μg/ml after 1, 3, and 5 days of treatment. PI staining and flow cytometer assay showed the effects of KR-12-a5 on the cell cycle. Compared to the control group, different concentrations of KR-12-a5 exhibited no significant influences on cell cycle progression of HBMSCs after 48 h of incubation (**Figure 1B**). Annexin V staining results showed the potential apoptotic effects of KR-12-a5. **Figure 1C** showed that the various concentrations of KR-12-a5 did not induce the early apoptosis of HBMSCs when compared to the control group. And the apoptotic states among these four tested concentrations had no significant difference.

### KR-12-a5 Reverses the Adverse Effects of LPS on HBMSC Osteogenic Differentiation

**Figure 2:**
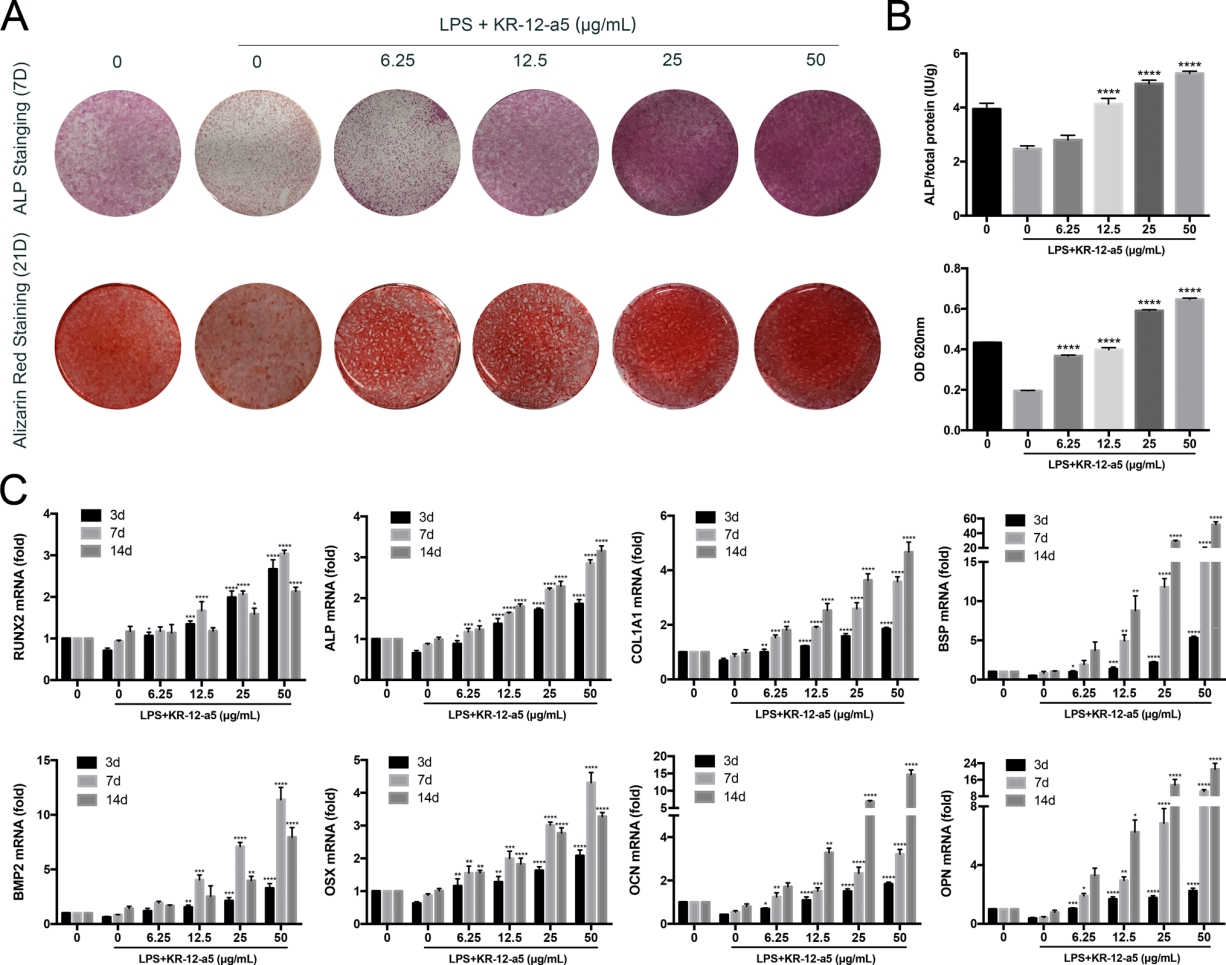
The effect of KR-12-a5 on LPS-induced osteogenic differentiation of HBMSCs. Cells were pre-treated with LPS (1 μg/ml) for 24 h and then incubated with osteogenic differentiation medium with the indicated concentrations of KR-12-a5 (0–50 μg/ml). The LPS group was used as a control group when performing statistical analysis. **(A)** Entire plate views of alkaline phosphatase (ALP) staining at 7 days and Alizarin red staining at 21 days. **(B)** Quantitative evaluation of ALP activity and Alizarin red staining results (****P < 0.0001). **(C)** Runx2, ALP, COL1A1, BSP, BMP2, OSX, OCN, and OPN mRNAs were subjected to real-time PCR analysis at 3, 7, and 14 days. The expression levels were normalized to that of GAPDH (*P < 0.05, **P < 0.01, ***P < 0.001, ****P < 0.0001).

To determine the influence of KR-12-a5 on LPS-induced HBMSC osteogenic differentiation, ALP and Alizarin Red staining and quantitative tests were performed. After LPS stimulation, ALP and Alizarin Red staining intensities were significantly reduced in HBMSCs. However, adding different concentrations of KR-12-a5 after LPS stimulation greatly increased the ALP and Alizarin Red staining intensities when compared to that of LPS alone. These increases were proportional to KR-12-a5 concentrations. When KR-12-a5 concentrations reached 25 μg/ml or more, HBMSC staining intensities exceeded the negative control group without LPS (**Figure 2A**). Similar results were observed in ALP quantitative tests (**Figure 2B**). Furthermore, the level of osteogenic differentiation genes (RUNX2, ALP, COL1A1, BSP, BMP2, OSX, OPN, and OCN) was determined by RT-qPCR. Under LPS-pretreatment conditions, the mRNA levels of RUNX2 increased in a concentration-dependent manner after 3 days of KR-12-a5 treatment. After 7 days, the level of ALP, COL1A1, BSP, and BMP2 expression in the KR-12-a5-treated groups showed obvious enhancement when compared to the LPS-stimulated group. Meanwhile, the OSX, OCN, and OPN gene levels also started to enhance at day 7. By the 14th day of KR-12-a5 treatment, the OSX, OCN, and OPN levels increased significantly (**Figure 2C**).

### KR-12-a5 Inhibits LPS-Activated P38 MAPK Signaling in HBMSCs

**Figure 3:**
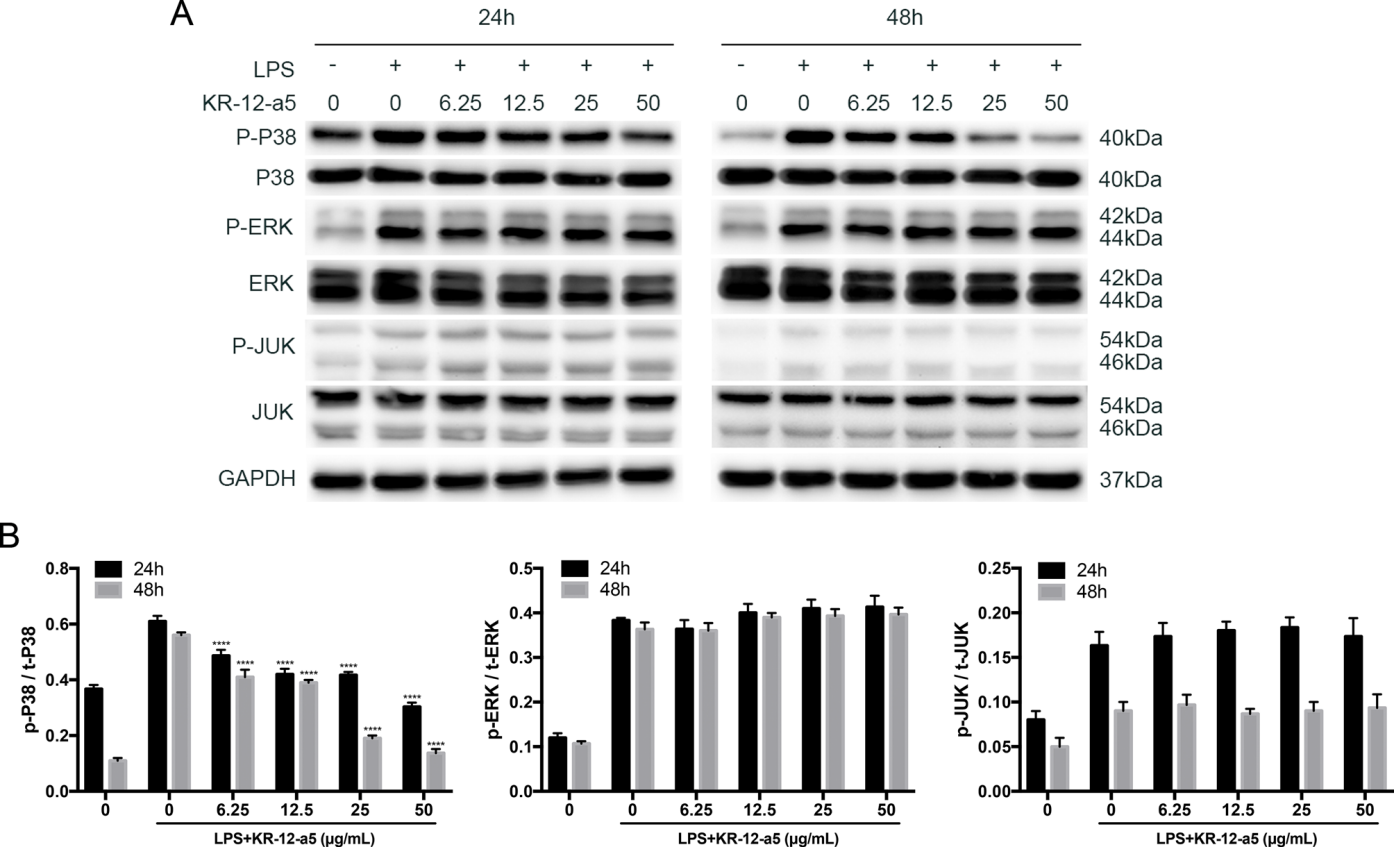
The effect of KR-12-a5 on MAPKs signaling after LPS stimulation in HBMSCs. The LPS group was used as a control group when performing statistical analysis. **(A)** The levels of phosphorylated and overall P38, JNK, and ERK at 24 and 48 h were examined *via* Western blotting. **(B)** The quantitative assay of p-P38/t-P38, p-REK/t-ERK, and p-JNK/t-JNK (****P < 0.0001).

To determine whether different doses of KR-12-a5 affected LPS-stimulated HBMSC differentiation *via* the MAPK signaling pathways, we investigated MAPK phosphorylation levels using Western blot analysis (**Figure 3A**) and quantified the resulting data (**Figure 3B**). LPS caused a strong activation of MAPK signaling when compared to the negative control group. After 24 h of KR-12-a5 treatment, P38 phosphorylation levels were weakened to varying degrees, and its phosphorylation levels negatively correlated with KR-12-a5 concentrations. However, ERK and c-Jun N-terminal kinase (JNK) phosphorylation levels did not change significantly with the addition of KR-12-a5. Furthermore, P38 phosphorylation levels were even more significantly reduced after 48 h of KR-12-a5 treatment. These results suggest that KR-12-a5 may affect HBMSC osteogenesis *via* P38 MAPK signaling.

### Similar Effects When Inhibiting P38 MAPK Signaling and Adding KR-12-a5

**Figure 4:**
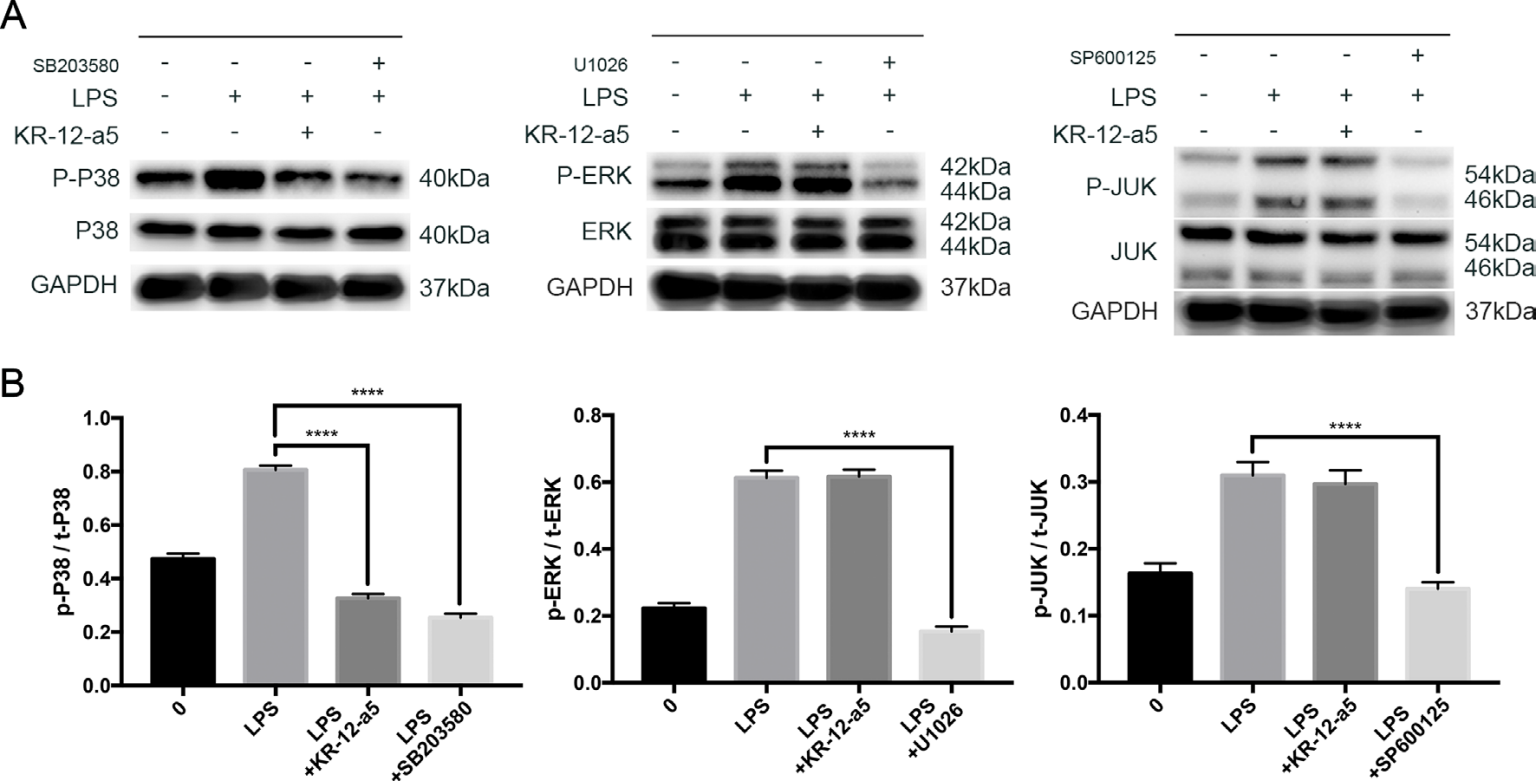
The comparison of inhibitory effect on MAPKs signaling between KR-12-a5 and MAPKs inhibitors. **(A)** The levels of phosphorylated and overall P38, JNK, and ERK at 24 h were examined *via* Western blotting. **(B)** The quantitative assay of p-P38/t-P38, p-REK/t-ERK, p-JNK/t-JNK (****P < 0.0001).

To further clarify the role of MAPK in the therapeutic effect of KR-12-a5, we blocked the MAPK signaling pathways using P38 (SB203580), ERK (U0126), and JNK (SP600125) inhibitors (all at 10 μM, Selleck, USA) to make a comparison with KR-12-a5 treatment. Western blot analysis was performed to determine MAPK phosphorylation levels after a 24-h treatment. The results showed that the inhibitory effect of KR-12-a5 on P38 protein phosphorylation was similar to that of the P38 inhibitor, SB203580. However, KR-12-a5 did not produce similar inhibitory effects as the ERK (U0126) and JNK (SP600125) inhibitors (**Figure 4A**). The quantified Western blot results are shown in **Figure 4B**.

### KR-12-a5 Reverses LPS-Induced Inhibition of BMP/Smad Signaling in HBMSCs

**Figure 5:**
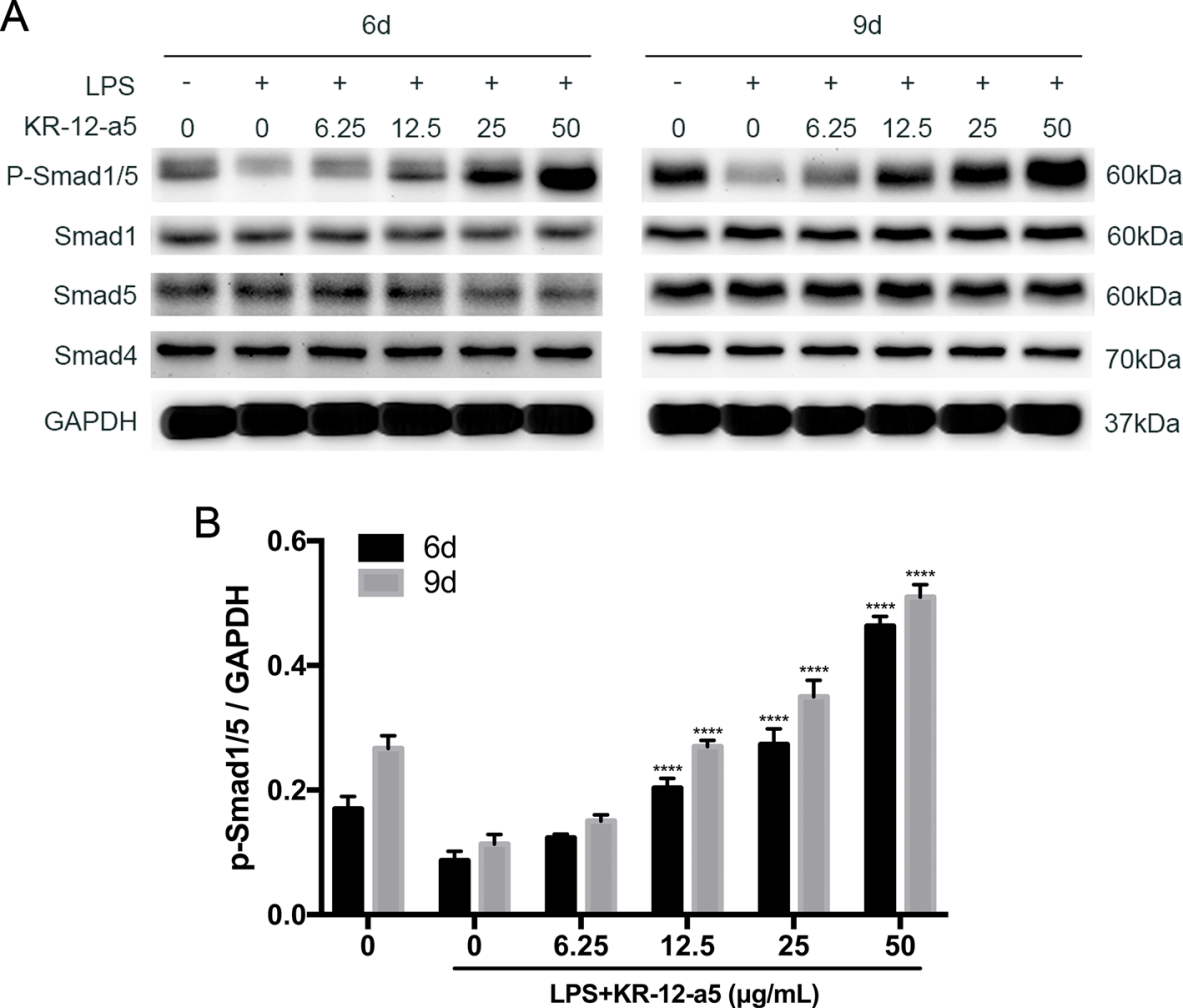
The effect of KR-12-a5 on BMP/Smad signaling after LPS stimulation in HBMSCs. The LPS group was used as a control group when performing statistical analysis. **(A)** The levels of phosphorylated Smad1/5, overall Smad1, Sma5, and Smad4 at 6 and 9 days were examined *via* Western blotting. **(B)** The quantitative assay of P-Smad1/5/GAPDH (****P < 0.0001).

Western blot analysis showed the effect of KR-12-a5 on LPS-stimulated BMP/Smad signaling. As shown in **Figure 2C**, KR-12-a5 significantly activated the LPS-inhibited BMP2 gene expression. The downstream components of the Smad signaling were also examined during osteogenic differentiation process of HBMSCs. We found that Pre-LPS stimulation significantly inhibited Smad1/5 phosphorylation at day 6, whereas different doses of KR-12-a5 (6.25–50 μg/ml) reversed the effects of LPS and significantly increased Smad1/5 phosphorylation levels in a dose-dependent manner. At day 9, Smad1/5 phosphorylation levels showed a slight increase in response to KR-12-a5 (**Figure 5A**). The quantified data are shown in **Figure 5B**. We also found that Smad4 expression levels remained unchanged after LPS or LPS/KR-12-a5 stimulation (**Figure 5A**). Therefore, these findings suggested that KR-12-a5 reversed the LPS-mediated inhibition of BMP/Smad signaling in HBMSCs and increased Smad1/5 phosphorylation levels in a dose-dependent manner.

### BMP/Smad Inhibition Suppresses the Therapeutic Effect of KR-12-a5

**Figure 6:**
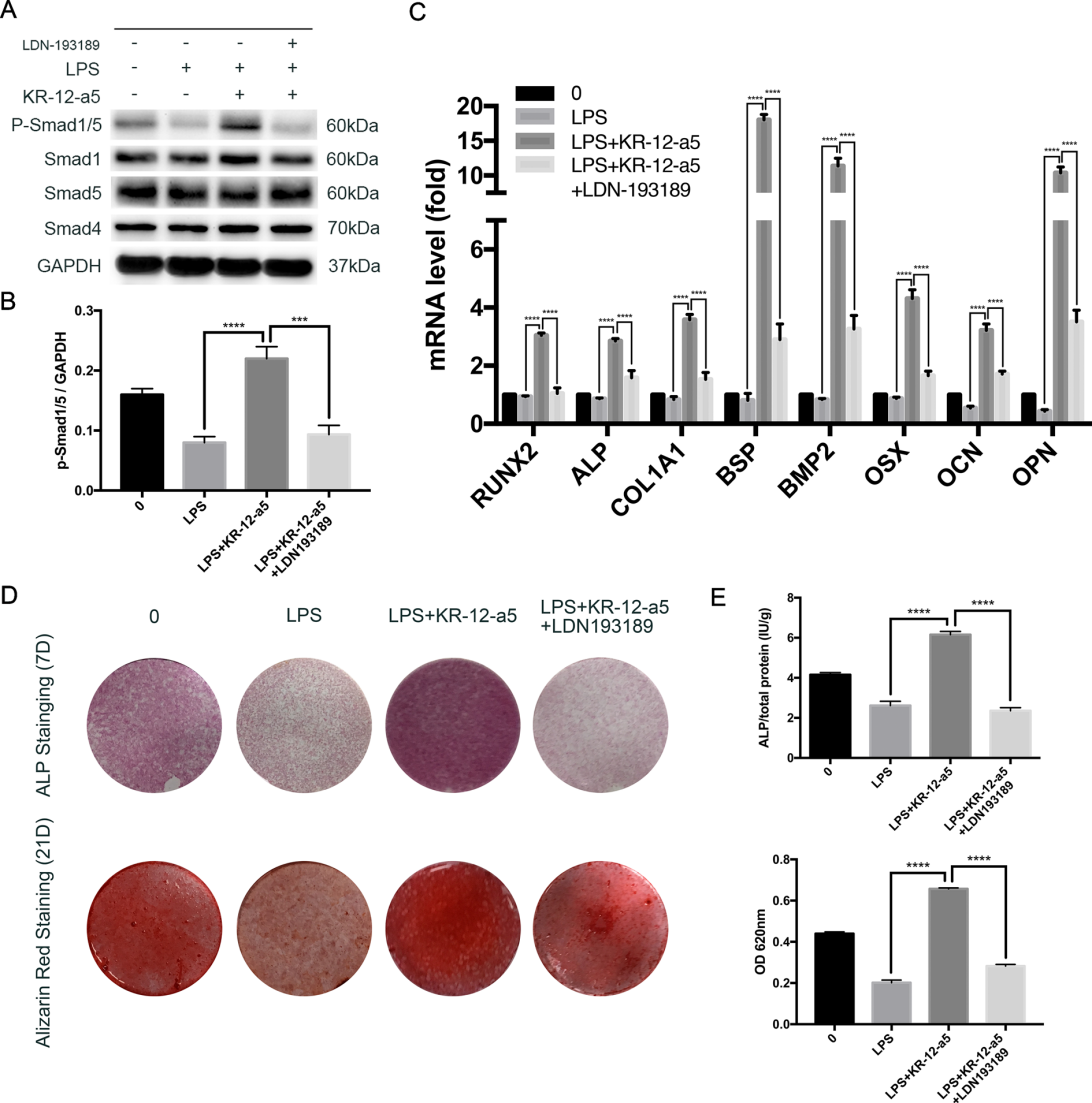
The effect of TGF-β/Smad inhibitors on the osteogenic differentiation of HBMSCs. After previous LPS processing, HBMSCs were treated with osteogenic differentiation medium in the presence of KR-12-a5 (50 μg/ml) along with an inhibitor of TGF-β/Smad (LDN193189). **(A)** Western blot analysis results for the levels of phosphorylated Smad1/5, overall Smad1, Sma5, and Smad4 at 6 days. **(B)** The quantitative assay of P-Smad1/5/GAPDH (***P < 0.001, ****P < 0.0001). **(C)** Runx2, ALP, COL1A1, BSP, BMP2, OSX, OCN, and OPN mRNAs were subjected to quantitative real-time PCR analysis at 7 days. The expression levels were normalized to that of GAPDH (****P < 0.0001). **(D)** Entire plate views of alkaline phosphatase (ALP) staining at 7 days and Alizarin red staining at 21 days. **(E)** Quantitative evaluation of ALP activity and Alizarin red staining results (****P < 0.0001).

To further evaluate the role of BMP/Smad signaling in osteogenic differentiation under KR-12-a5 treatment, a TGF-β/Smad inhibitor (LDN193189, 0.2 μM, Selleck, USA) was used to block the BMP/Smad signaling. LDN193189 pre-stimulated for 1 h, and then stimulated with the KR-12-a5 until the time of detection. The results of Western blot analysis showed the Smad phosphorylation levels after 6 days (**Figure 6A**). The addition of inhibitors reduced Smad1/5 phosphorylation just like the high KR-12-a5 concentration treatments to similar levels observed in the LPS alone treatment. The quantified results of Western blot were shown in **Figure 6B**. As shown in **Figure 6D and E**, ALP and mineralization assay in HBMSCs was inhibited by the blocking of BMP/Smad signaling. These results confirmed that BMP/Smad signaling played a therapeutic role in reversing LPS-suppressed osteogenic differentiation. RT-qPCR results further supported this observation. Blocking the BMP/Smad signaling pathway suppressed the expression of osteoblast marker genes (RUNX2, ALP, COL1A1, BSP, BMP2, OSX, OCN, and OPN) at day 7 (**Figure 6C**).

### KR-12-a5 Inhibits ROS Generation and Increases SOD Activity in LPS-Induced HBMSCs

**Figure 7:**
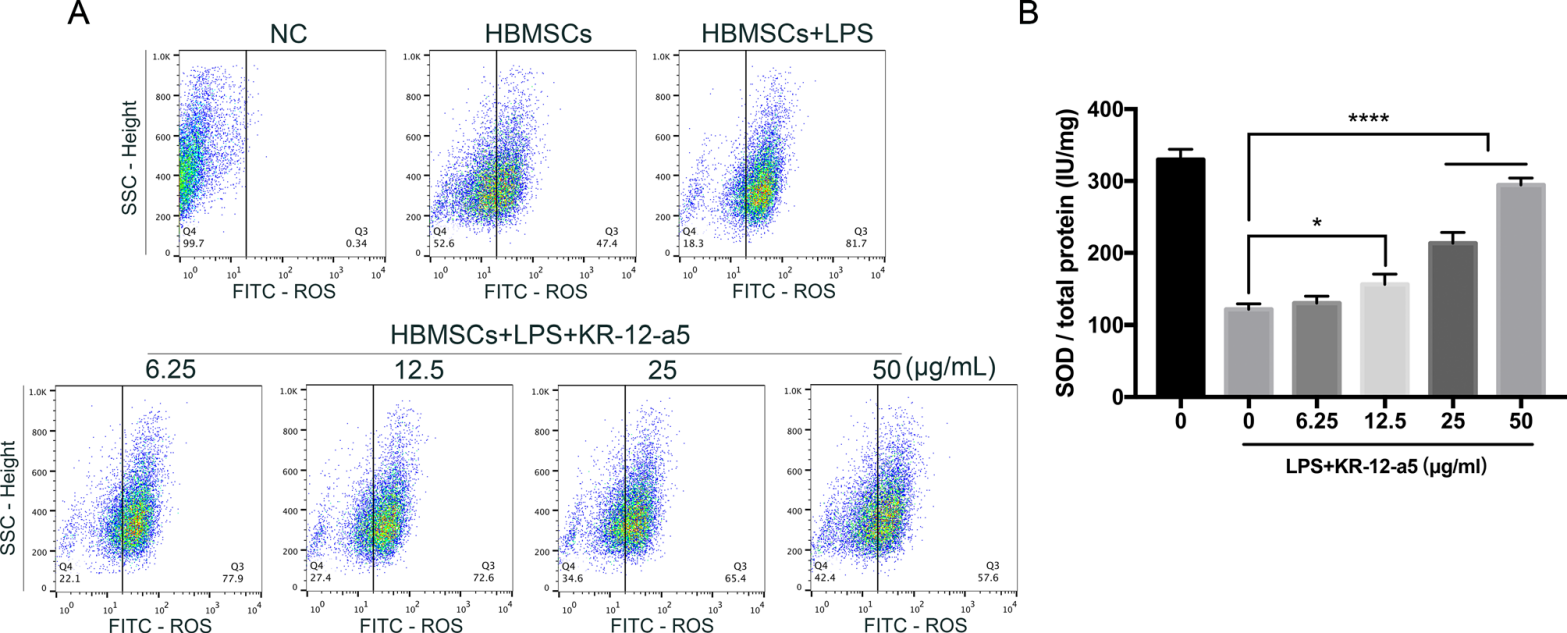
The levels of ROS generation and SOD activity in LPS-induced HBMSCs after treated with KR-12-a5. The LPS group was used as a control group when performing statistical analysis. **(A)** The ROS level (intensity of DCFDA fluorescence) at 48 h. **(B)** SOD activity at 7 days (*P < 0.05, ****P < 0.0001).

The levels of ROS and SOD activity in LPS-induced HBMSCs were measured to determine the antioxidant properties of KR-12-a5. After LPS induction, the ROS levels in HBMSCs increased significantly, while activity of SOD decreased. However, after 48 h or 7 days of KR-12-a5 treatment, the level of ROS was downregulated (**Figure 7A**) and SOD activity was partially upregulated (**Figure 7B**) when compared to the LPS-induced groups. The extent of these effects was also related to KR-12-a5 concentrations.

### KR-12-a5 Reverses LPS-Induced Bone Erosion *In Vivo*

**Figure 8:**
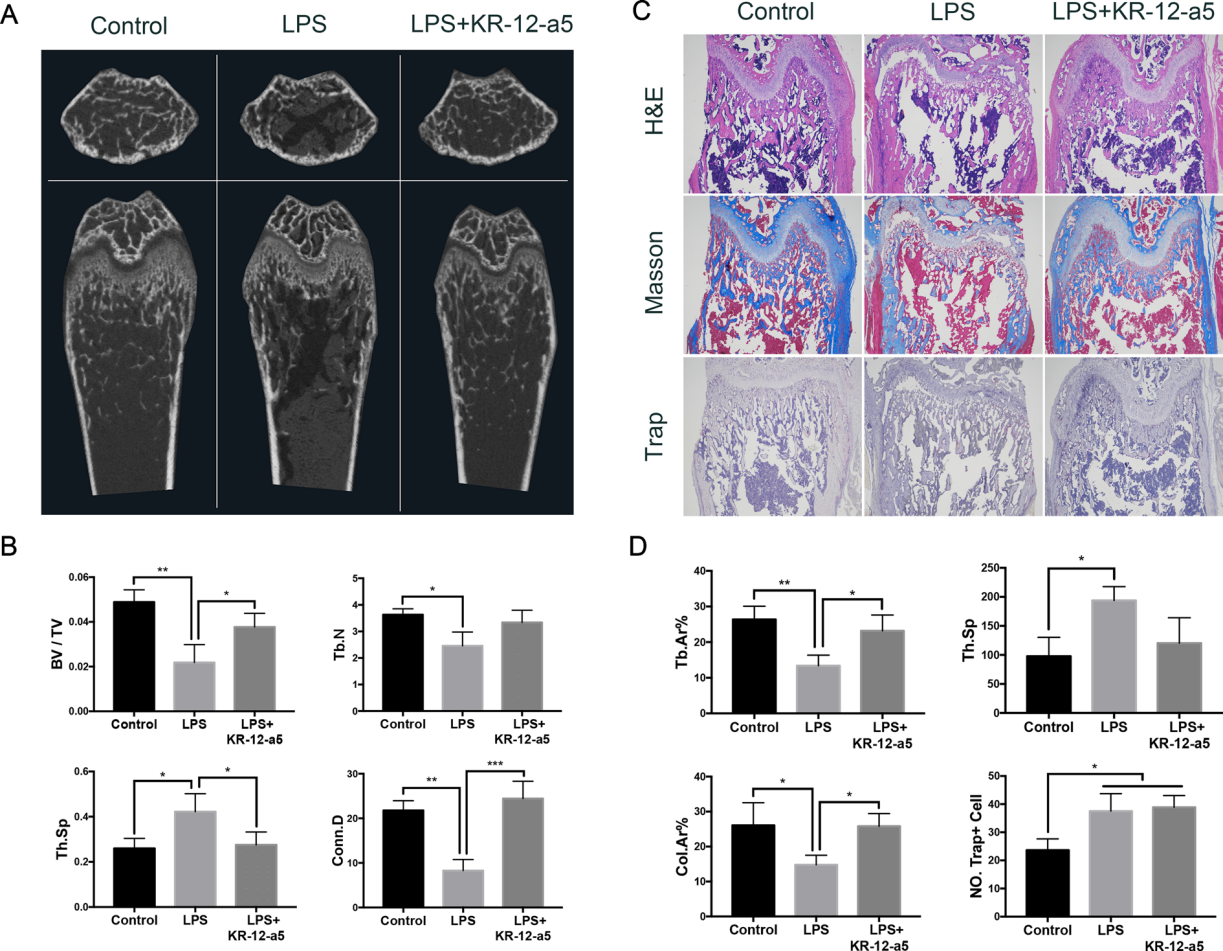
The effect of KR-12-a5 on LPS-induced bone erosion *in vivo*. **(A)** Scanned images from micro-CT. **(B)** The bone volume/tissue volume (BV/TV), Trabecular number (Tb.N), Trabecular separation (Tb.Sp), and connectivity density (Conn. D) from the micro-CT data (*P < 0.05, **P < 0.01, ***P < 0.001). **(C)** Representative images of decalcified bone stained with H&E, Masson, and TRAP. Bars represent 500 μm. **(D)** Quantitative analyses of Trabecular area percentage (Tb.Ar%), Tb.Sp, Collagen fiber area percentage (Col.Ar%), and number of TRAP-positive cells per unit area (NO. Trap+ Cell) (*P < 0.05, **P < 0.01).

The micro-CT results showed the femoral bone mass of the mice after 9 days of treatment. As shown in **Figure 8A**, LPS decreased trabecular bone mass, which was inhibited by KR-12-a5. Micro-CT analysis of the femurs revealed the LPS-induced erosion in bone mass by calculating BV/TV, Tb.N, Tb.Sp, and Conn.D in LPS-treated mice, which was reversed by KR-12-a5 (**Figure 8B**).

Histological analyses further confirmed the protective effect of KR-12-a5 in LPS-induced osteolysis. Consistent with the results of the micro-CT, H&E staining results showed an increased bone formation in the LPS+KR-12-a5 group when compared to the LPS group. The results also showed positive staining of collagen in the femurs and demonstrated that their expression was significantly reduced after LPS stimulation. After KR-12-a5 administration, collagen expression was greatly elevated (**Figure 8C and D**). These results demonstrated the therapeutic effects of KR-12-a5 against LPS-induced inflammatory bone erosion *in vivo*. To further explore if the reduced bone loss was due to the decreased osteoclastogenesis, we checked the number of osteoclasts in the femurs. Trap staining showed no significant differences in terms of mature osteoclast number between the LPS group and the LPS+KR-12-a5 group (**Figure 8C**). Quantitative analysis results show that the number of Trap-positive cells per unit area was not significantly different between the LPS group and LPS+KR-12-a5 group (**Figure 8D**).

## Discussion

This study investigated the role of KR-12-a5 treatment in LPS-induced inflammatory environments and the BMP/Smad and MAPK signaling pathways in the regulation of HBMSC osteogenic differentiation. An inflammatory microenvironment was mimicked by pre-treating HBMSCs with LPS and different concentrations of the antimicrobial peptide, KR-12-a5, were used for treatment. Our novel findings are as follows: 1) KR-12-a5 does not adversely affect HBMSCs within a therapeutically effective concentration range; 2) KR-12-a5 has a dose-dependent role in reversing the LPS-induced osteogenic inhibition of HBMSCs; 3) KR-12-a5 reverses the LPS-induced inhibition of BMP/Smad signaling and inhibits the LPS-activated P38 MAPK signaling pathway; 4) KR-12-a5 has a positive therapeutic effect on the LPS-induced increase in oxidative stress; and 5) KR-12-a5 has a therapeutic effect on LPS-induced osteolysis in mice.

The potential cytotoxic effects of KR-12-a5 were investigated before examining its effects on LPS-induced osteogenic differentiation. The concentration range of 0–64 μg/ml was not cytotoxic to HBMSCs, but it could effectively kill various pathogens. This concentration range was much larger than KR-12-a5’s minimum inhibitory concentration (MIC) for clinically pathogenic bacteria such as *Pseudomonas aeruginosa* (12.5 μg/ml), *Salmonella typhimurium* (3.125 μg/ml), *Bacillus subtilis* (12.5 μg/ml), *Staphylococcus epidermidis* (6.25 μg/ml), *Staphylococcus aureus* (6.25 μg/ml), and *Escherichia coli* (12.5 μg/ml) (Kim et al., 2017). Therefore, the experimental concentrations of KR-12-a5 in subsequent experiments were set to four concentrations between 6.25 and 50 μg/ml. Cell cycle assay demonstrated that KR-12-a5 did not affect cell cycle progression, indicating that it did not significantly influence proliferation at this concentration range. KR-12-a5 also had no effect on apoptosis. All these results indicate that KR-12-a5 possesses good biocompatibility and excellent antibacterial properties at the same time, which is very similar to KR-12 (Jacob et al., 2013; Li et al., 2018) and provides the basis for its subsequent therapeutic effects.

A variety of inflammatory factors and bacterial components are commonly known to act as negative modulators in HBMSC osteogenesis. Several studies have demonstrated that a cell wall component of gram-negative bacteria, LPS, inhibits MSC osteogenesis or suppresses osteoblast-related gene expression (Kadono et al., 1999; Bandow et al., 2010; Ochi et al., 2010; Xing et al., 2010). Surprisingly, our results demonstrate that the LPS-induced adverse effects on HBMSC osteogenic and mineralization potential can be reversed by KR-12-a5 in a concentration-dependent manner. These adverse effects can be completely reversed and even exhibit stronger osteogenic potential than non LPS-stimulated groups when the concentration of KR-12-a5 reaches 25 μg/ml or more. We speculate that the LPS-activated inflammatory response disrupts the local osteogenic microenvironment, which is very important for the differentiation of HBMSCs into osteoblasts. However, the addition of KR-12-a5 improves the local microenvironment, making it suitable for osteogenic differentiation. Therefore, regulating the function of resident stem cells by improving the local inflammatory microenvironment may have important clinical significance in improving the therapeutic effect of infection-associated osteolysis.

Several downstream signaling pathways can be activated by LPS, including Akt, NF-κB, and MAPK signaling (Jung et al., 2009; Guo et al., 2014; Guo et al., 2015). Among them, MAPK signaling is regulated by a characteristic phosphorylation system in which a series of three protein kinases phosphorylate and activate one another. P38, ERK, and JNK pathways together constitute this signaling (Johnson and Lapadat, 2002). In this study, we found that the P38/MAPK, ERK/MAPK, and JNK/MAPK pathways were phosphorylated after LPS stimulation, but only P38 phosphorylation levels could be reduced after KR-12-a5 treatment. Compared to the P38, ERK, and JNK MAPK pathway inhibitors, KR-12-a5 only exhibited similar effects as the P38 inhibitor (SB203580), and not those of the JNK (SP600125) and ERK (U1026) inhibitors. This suggests that KR-12-a5 may control the LPS-induced inflammatory response *via* P38 MAPK signaling, which serves as a molecular target for improving the inflammatory environment in chronic osteomyelitis.

BMP/Smad signaling plays important roles in regulating osteoblast differentiation (Chen et al., 2012), which can be influenced by many inflammatory factors such as LPS and TNF-α (Huang et al., 2014; Loebel et al., 2017). It also has been reported that BMP/Smad signaling can function independently of RUNX2, whereas BMP2-induced OSX expression is mainly mediated by Dlx5 and not RUNX2 (Lee et al., 2003). Our findings show that KR-12-a5 can reactivate LPS-inhibited Smad1/5 phosphorylation levels in a dose-dependent manner. Osteoblast maker genes such as RUNX2, ALP, COL1A1, BSP, BMP2, OSX, OCN, and OPN can also be enhanced by this reactivation. KR-12-a5-mediated Smad1/5 activation can be blocked by the TGFβ/Smad antagonist, LDN193189, which further leads to a decrease in ALP, mineralization, and osteogenic marker genes in HBMSCs. Therefore, while controlling the inflammatory response during infection, attention to the role of the BMP/Smad pathway during local bone regeneration may have a positive effect in treating infection-associated osteolysis.

Furthermore, studies have shown that osteomyelitis elevates local oxidative stress levels, which may severely distort the osteogenesis microenvironment (Tekin Koruk et al., 2012; Grbic et al., 2014). LPS-induced oxidative stress plays an important role in the inflammatory processes leading to alveolar bone loss in periodontitis (Kose et al., 2016). The excessive ROS production severely damages DNA, proteins, and lipids, which affects not only cell proliferation but also differentiation (Moriwaki et al., 2014; Huang et al., 2015). In this study, assay of ROS production and SOD activity showed that the LPS-induced oxidative injury in HBMSCs was inhibited by KR-12-a5 treatment. The findings clearly demonstrated that KR-12-a5 is capable of not only inducing a preferential environment to drive osteogenic differentiation in LPS-induced HBMSCs, but also of reversing LPS-induced oxidative stress due to its antioxidant properties. All this provides a new direction for the treatment of inflammatory conditions in osteomyelitis.

A model of LPS-induced inflammatory bone loss was successfully set up by stimulating distinct bone erosion in the spongiosa of murine femurs. As expected, KR-12-a5 treatment significantly mitigated the severity of the bone erosion in the LPS-treated mouse model, which was assessed by an increase in BV/TV, Tb.N, Tb.Sp, and Conn.D *via* micro-CT scanning. Similarly, histological analyses further confirmed that KR-12-a5 treatment reversed LPS-induced inflammatory bone loss, accompanied with elevated collagen expression. It was worth mentioning that the number of Trap+ cells activated by LPS did not decrease with the treatment of KR-12-a5 *in vivo*. Thus, we speculated that the reversed femur bone loss was due to the elevated redifferentiation of the HBMSCs and that KR-12-a5 had the therapeutic potential to treat inflammatory bone erosion *in vivo*. Nevertheless, the detailed mechanism still needs further investigation as the *in vivo* LPS-induced osteolysis mechanism is complicated and not completely understood.

In spite of these novel findings in our study, there are several limitations that merit discussion. Firstly, the interaction between osteogenesis and bone resorption is complex and osteoclastic bone resorption also significantly affects osteolysis treatment. In this paper, we mainly concentrated on the effect and mechanism of KR-12-a5 with respect to osteoblastic bone formation in the present study. The *in vivo* study indicated that the intraperitoneal administration of KR-12-a5 strongly reversed bone loss and increased the ALP and collagen content in the femurs. Since the histological staining results showed that KR-12-a5 cannot reduce the number of Trap-positive cells, further studies are needed to address its effect on osteoclasts *in vitro*. Secondly, other potential mechanisms behind the therapeutic effects of KR-12-a5 deserve further exploration since there are many signaling pathways related to osteogenic differentiation and LPS stimulation in HBMSCs.

## Conclusion

In summary, the findings of this study demonstrated that KR-12-a5 reversed the osteogenic differentiation potential of HBMSCs *in vitro* and LPS-induced inflammatory bone erosion *in vivo*. We clarified that this occurred by suppressing the P38 MAPK and reactivating the BMP/Smad signaling pathways. Moreover, the protective effects of KR-12-a5 against LPS-induced oxidative stress in HBMSCs were found under osteogenesis conditions. These results suggested that KR-12-a5 might serve as a potential therapeutic agent for osteomyelitis-related osteolysis.

## Data Availability Statement

The raw data supporting the conclusions of this manuscript will be made available by the authors, without undue reservation, to any qualified researcher.

## Ethics Statement

All experiments were performed in accordance with the guidelines “Ethical review methods for biomedical research involving people” and “Guidance for Animal Research,” and approved by the ethics committee at Renji Hospital, Shanghai Jiaotong University School of Medicine. The registration number of the ethics committee is SHNHEA[2015]75. Informed consents were obtained from human participants of this study.

## Author Contributions

HL, SZ, BN, and TL carried out the experiments. HL wrote the manuscript. BY and XQ designed the experiments. BY revised the manuscript. All authors reviewed the manuscript.

## Funding

This research was supported by the National Natural Science Foundation of China (81472119 and 81672196) and Shanghai municipal education commission—Gaofeng clinical medicine grant support (20161423).

## Conflict of Interest Statement

The authors declare that the research was conducted in the absence of any commercial or financial relationships that could be construed as a potential conflict of interest.

## Abbreviations

HBMSCs, human bone marrow mesenchymal stem cells; LPS, lipopolysaccharide; ERK1/2, extracellular signal-regulated kinase 1/2; MAPK, mitogen-activated protein kinase; p38, p38 kinase; JNK, c-Jun N-terminal kinase; GAPDH, glyceraldehyde-3-phosphate dehydrogenase; Runx2, Runt-related transcription factor 2; ALP, alkaline phosphatase; COL1A1, type I collagen; BSP, bone sialoprotein; OCN, osteocalcin; OPN, osteopontin; OSX, osterix; BMP, bone morphogenetic protein; ROS, reactive oxygen species; SOD, superoxide dismutaset.

## Supplementary Material

Figure S1 Surface marker analysis of HBMSCs by flow cytometry.

## References

[B1] Alcayaga-Miranda F. Cuenca J. Khoury M. (2017). Antimicrobial activity of mesenchymal stem cells: current status and new perspectives of antimicrobial peptide-based therapies. Front. Immunol. 8, 339. 10.3389/fimmu.2017.00339 28424688 PMC5371613

[B2] Bandow K. Maeda A. Kakimoto K. Kusuyama J. Shamoto M. Ohnishi T. (2010). Molecular mechanisms of the inhibitory effect of lipopolysaccharide (LPS) on osteoblast differentiation. Biochem. Biophys. Res. Commun. 402, 755–761. 10.1016/j.bbrc.2010.10.103 21036155

[B3] Bernard L. Dinh A. Ghout I. Simo D. Zeller V. Issartel B. (2015). Antibiotic treatment for 6 weeks versus 12 weeks in patients with pyogenic vertebral osteomyelitis: an open-label, non-inferiority, randomised, controlled trial. Lancet 385, 875–882. 10.1016/S0140-6736(14)61233-2 25468170

[B4] Bielby R. C. Boccaccini A. R. Polak J. M. Buttery L. D. (2004). *In vitro* differentiation and *in vivo* mineralization of osteogenic cells derived from human embryonic stem cells. Tissue Eng. 10, 1518–1525. 10.1089/ten.2004.10.1518 15588411

[B5] Boman H. G. (1995). Peptide antibiotics and their role in innate immunity. Annu. Rev. Immunol. 13, 61–92. 10.1146/annurev.iy.13.040195.000425 7612236

[B6] Bowdish D. M. Davidson D. J. Speert D. P. Hancock R. E. (2004). The human cationic peptide LL-37 induces activation of the extracellular signal-regulated kinase and p38 kinase pathways in primary human monocytes. J. Immunol. 172, 3758–3765. 10.4049/jimmunol.172.6.3758 15004180

[B7] Bucki R. Leszczynska K. Namiot A. Sokolowski W. (2010). Cathelicidin LL-37: a multitask antimicrobial peptide. Arch. Immunol. Ther. Exp. (Warsz). 58, 15–25. 10.1007/s00005-009-0057-2 20049649

[B8] Carvalho V. C. Oliveira P. R. Dal-Paz K. Paula A. P. Felix Cda S. Lima A. L. (2012). Gram-negative osteomyelitis: clinical and microbiological profile. Braz. J. Infect. Dis. 16, 63–67. 10.1590/S1413-86702012000100011 22358358

[B9] Chen G. Deng C. Li Y. P. (2012). TGF-beta and BMP signaling in osteoblast differentiation and bone formation. Int. J. Biol. Sci. 8, 272–288. 10.7150/ijbs.2929 22298955 PMC3269610

[B10] Chiang C. Y. Kyritsis G. Graves D. T. Amar S. (1999). Interleukin-1 and tumor necrosis factor activities partially account for calvarial bone resorption induced by local injection of lipopolysaccharide. Infect. Immun. 67, 4231–4236. 10417196 10.1128/iai.67.8.4231-4236.1999 PMC96729

[B11] Ciornei C. D. Sigurdardottir T. Schmidtchen A. Bodelsson M. (2005). Antimicrobial and chemoattractant activity, lipopolysaccharide neutralization, cytotoxicity, and inhibition by serum of analogs of human cathelicidin LL-37. Antimicrob. Agents Chemother. 49, 2845–2850. 10.1128/AAC.49.7.2845-2850.2005 15980359 PMC1168709

[B12] Dorschner R. A. Pestonjamasp V. K. Tamakuwala S. Ohtake T. Rudisill J. Nizet V. (2001). Cutaneous injury induces the release of cathelicidin anti-microbial peptides active against group A Streptococcus. J. Invest. Dermatol. 117, 91–97. 10.1046/j.1523-1747.2001.01340.x 11442754

[B13] Franceschi R. T. Xiao G. (2003). Regulation of the osteoblast-specific transcription factor, Runx2: responsiveness to multiple signal transduction pathways. J. Cell Biochem. 88, 446–454. 10.1002/jcb.10369 12532321

[B14] Frohm M. Agerberth B. Ahangari G. Stahle-Backdahl M. Liden S. Wigzell H. (1997). The expression of the gene coding for the antibacterial peptide LL-37 is induced in human keratinocytes during inflammatory disorders. J. Biol. Chem. 272, 15258–15263. 10.1074/jbc.272.24.15258 9182550

[B15] Grbic R. Miric D. J. Kisic B. Popovic L. Nestorovic V. Vasic A. (2014). Sequential analysis of oxidative stress markers and vitamin C status in acute bacterial osteomyelitis. Mediators Inflamm. 2014, 975061. 10.1155/2014/975061 25180026 PMC4142778

[B16] Guo C. Yuan L. Wang J. G. Wang F. Yang X. K. Zhang F. H. (2014). Lipopolysaccharide (LPS) induces the apoptosis and inhibits osteoblast differentiation through JNK pathway in MC3T3-E1 cells. Inflammation 37, 621–631. 10.1007/s10753-013-9778-9 24272171

[B17] Guo C. Wang S. L. Xu S. T. Wang J. G. Song G. H. (2015). SP600125 reduces lipopolysaccharide-induced apoptosis and restores the early-stage differentiation of osteoblasts inhibited by LPS through the MAPK pathway in MC3T3-E1 cells. Int. J. Mol. Med. 35, 1427–1434. 10.3892/ijmm.2015.2130 25760015

[B18] Huang R. L. Yuan Y. Zou G. M. Liu G. Tu J. Li Q. (2014). LPS-stimulated inflammatory environment inhibits BMP-2-induced osteoblastic differentiation through crosstalk between TLR4/MyD88/NF-kappaB and BMP/Smad signaling. Stem Cells Dev. 23, 277–289. 10.1089/scd.2013.0345 24050190 PMC3904516

[B19] Huang Q. Gao B. Wang L. Zhang H. Y. Li X. J. Shi J. (2015). Ophiopogonin D: a new herbal agent against osteoporosis. Bone 74, 18–28. 10.1016/j.bone.2015.01.002 25582622

[B20] Islam S. Hassan F. Tumurkhuu G. Dagvadorj J. Koide N. Naiki Y. (2007). Bacterial lipopolysaccharide induces osteoclast formation in RAW 264.7 macrophage cells. Biochem. Biophys. Res. Commun. 360, 346–351. 10.1016/j.bbrc.2007.06.023 17597583

[B21] Itoh K. Udagawa N. Kobayashi K. Suda K. Li X. Takami M. (2003). Lipopolysaccharide promotes the survival of osteoclasts *via* Toll-like receptor 4, but cytokine production of osteoclasts in response to lipopolysaccharide is different from that of macrophages. J. Immunol. 170, 3688–3695. 10.4049/jimmunol.170.7.3688 12646634

[B22] Jacob B. Park I. S. Bang J. K. Shin S. Y. (2013). Short KR-12 analogs designed from human cathelicidin LL-37 possessing both antimicrobial and antiendotoxic activities without mammalian cell toxicity. J. Pept. Sci. 19, 700–707. 10.1002/psc.2552 24105706

[B23] Johnson G. L. Lapadat R. (2002). Mitogen-activated protein kinase pathways mediated by ERK, JNK, and p38 protein kinases. Science 298, 1911–1912. 10.1126/science.1072682 12471242

[B24] Jung W. K. Park I. S. Park S. J. Yea S. S. Choi Y. H. Oh S. (2009). The 15-deoxy-Delta12,14-prostaglandin J2 inhibits LPS-stimulated AKT and NF-kappaB activation and suppresses interleukin-6 in osteoblast-like cells MC3T3E-1. Life Sci. 85, 46–53. 10.1016/j.lfs.2009.04.010 19409914

[B25] Kadono H. Kido J. Kataoka M. Yamauchi N. Nagata T. (1999). Inhibition of osteoblastic cell differentiation by lipopolysaccharide extract from Porphyromonas gingivalis. Infect. Immun. 67, 2841–2846. 10338489 10.1128/iai.67.6.2841-2846.1999 PMC96590

[B26] Karner E. Backesjo C. M. Cedervall J. Sugars R. V. Ahrlund-Richter L. Wendel M. (2009). Dynamics of gene expression during bone matrix formation in osteogenic cultures derived from human embryonic stem cells *in vitro* . Biochim. Biophys. Acta 1790, 110–118. 10.1016/j.bbagen.2008.10.004 19007861

[B27] Kim K. J. Yeon J. T. Choi S. W. Moon S. H. Ryu B. J. Yu R. (2015). Decursin inhibits osteoclastogenesis by downregulating NFATc1 and blocking fusion of pre-osteoclasts. Bone 81, 208–216. 10.1016/j.bone.2015.07.023 26208796

[B28] Kim E. Y. Rajasekaran G. Shin S. Y. (2017). LL-37-derived short antimicrobial peptide KR-12-a5 and its d-amino acid substituted analogs with cell selectivity, anti-biofilm activity, synergistic effect with conventional antibiotics, and anti-inflammatory activity. Eur. J. Med. Chem. 136, 428–441. 10.1016/j.ejmech.2017.05.028 28525841

[B29] Kose O. Arabaci T. Kara A. Yemenoglu H. Kermen E. Kizildag A. (2016). Effects of melatonin on oxidative stress index and alveolar bone loss in diabetic rats with periodontitis. J. Periodontol. 87, e82–e90. 10.1902/jop.2016.150541 26832833

[B30] Krasnodembskaya A. Song Y. Fang X. Gupta N. Serikov V. Lee J. W. (2010). Antibacterial effect of human mesenchymal stem cells is mediated in part from secretion of the antimicrobial peptide LL-37. Stem Cells 28, 2229–2238. 10.1002/stem.544 20945332 PMC3293245

[B31] Le Clerc N. Verillaud B. Duet M. Guichard J. P. Herman P. Kania R. (2014). Skull base osteomyelitis: incidence of resistance, morbidity, and treatment strategy. Laryngoscope 124, 2013–2016. 10.1002/lary.24726 24752664

[B32] Lee M. H. Kwon T. G. Park H. S. Wozney J. M. Ryoo H. M. (2003). BMP-2-induced Osterix expression is mediated by Dlx5 but is independent of Runx2. Biochem. Biophys. Res. Commun. 309, 689–694. 10.1016/j.bbrc.2003.08.058 12963046

[B33] Lesse A. J. Freer C. Salata R. A. Francis J. B. Scheld W. M. (1987). Oral ciprofloxacin therapy for gram-negative bacillary osteomyelitis. Am. J. Med. 82, 247–253. 3555043

[B34] Li H. Zhang S. Nie B. E. Du Z. Long T. Yue B. (2018). The antimicrobial peptide KR-12 promotes the osteogenic differentiation of human bone marrow stem cells by stimulating BMP/SMAD signaling. RSC Adv. 8, 15547–15557. 10.1039/C8RA00750K PMC9080063 35539499

[B35] Loebel C. Czekanska E. M. Staudacher J. Salzmann G. Richards R. G. Alini M. (2017). The calcification potential of human MSCs can be enhanced by interleukin-1beta in osteogenic medium. J. Tissue Eng. Regen. Med. 11, 564–571. 10.1002/term.1950 25185894

[B36] Martindale J. L. Holbrook N. J. (2002). Cellular response to oxidative stress: signaling for suicide and survival. J. Cell Physiol. 192, 1–15. 10.1002/jcp.10119 12115731

[B37] Milhan N. V. M. De Barros P. P. De Lima Zutin E. A. De Oliveira F. E. Camargo C. H. R. Camargo S. E. A. (2017). The antimicrobial peptide LL-37 as a possible adjunct for the proliferation and differentiation of dental pulp stem cells. J. Endod. 43, 2048–2053. 10.1016/j.joen.2017.08.010 29033090

[B38] Mishra B. Epand R. F. Epand R. M. Wang G. (2013). Structural location determines functional roles of the basic amino acids of KR-12, the smallest antimicrobial peptide from human cathelicidin LL-37. RSC Adv. 3, 19560–19571. 10.1039/c3ra42599a PMC3844289 24307932

[B39] Moriwaki S. Suzuki K. Muramatsu M. Nomura A. Inoue F. Into T. (2014). Delphinidin, one of the major anthocyanidins, prevents bone loss through the inhibition of excessive osteoclastogenesis in osteoporosis model mice. PLoS One 9, e97177. 10.1371/journal.pone.0097177 24824988 PMC4019566

[B40] Mormann M. Thederan M. Nackchbandi I. Giese T. Wagner C. Hansch G. M. (2008). Lipopolysaccharides (LPS) induce the differentiation of human monocytes to osteoclasts in a tumour necrosis factor (TNF) alpha-dependent manner: a link between infection and pathological bone resorption. Mol. Immunol. 45, 3330–3337. 10.1016/j.molimm.2008.04.022 18538847

[B41] Mukherjee A. Wilson E. M. Rotwein P. (2010). Selective signaling by Akt2 promotes bone morphogenetic protein 2-mediated osteoblast differentiation. Mol. Cell Biol. 30, 1018–1027. 10.1128/MCB.01401-09 19995912 PMC2815574

[B42] Murakami M. Lopez-Garcia B. Braff M. Dorschner R. A. Gallo R. L. (2004). Postsecretory processing generates multiple cathelicidins for enhanced topical antimicrobial defense. J. Immunol. 172, 3070–3077. 10.4049/jimmunol.172.5.3070 14978112

[B43] Nagaoka I. Hirota S. Niyonsaba F. Hirata M. Adachi Y. Tamura H. (2002). Augmentation of the lipopolysaccharide-neutralizing activities of human cathelicidin CAP18/LL-37-derived antimicrobial peptides by replacement with hydrophobic and cationic amino acid residues. Clin. Diagn. Lab. Immunol. 9, 972–982. 10.1128/CDLI.9.5.972-982.2002 12204946 PMC120071

[B44] Ochi H. Hara Y. Tagawa M. Shinomiya K. Asou Y. (2010). The roles of TNFR1 in lipopolysaccharide-induced bone loss: dual effects of TNFR1 on bone metabolism *via* osteoclastogenesis and osteoblast survival. J. Orthop. Res. 28, 657–663. 10.1002/jor.21028 19890995

[B45] Orcel P. Feuga M. Bielakoff J. De Vernejoul M. C. (1993). Local bone injections of LPS and M-CSF increase bone resorption by different pathways *in vivo* in rats. Am. J. Physiol. 264, E391–E397. 10.1152/ajpendo.1993.264.3.E391 8460686

[B46] Ramos R. Silva J. P. Rodrigues A. C. Costa R. Guardao L. Schmitt F. (2011). Wound healing activity of the human antimicrobial peptide LL37. Peptides 32, 1469–1476. 10.1016/j.peptides.2011.06.005 21693141

[B47] Rider C. C. Mulloy B. (2010). Bone morphogenetic protein and growth differentiation factor cytokine families and their protein antagonists. Biochem. J. 429, 1–12. 10.1042/BJ20100305 20545624

[B48] Salasznyk R. M. Klees R. F. Hughlock M. K. Plopper G. E. (2004). ERK signaling pathways regulate the osteogenic differentiation of human mesenchymal stem cells on collagen I and vitronectin. Cell Commun. Adhes. 11, 137–153. 10.1080/15419060500242836 16194881

[B49] Scott M. G. Vreugdenhil A. C. Buurman W. A. Hancock R. E. Gold M. R. (2000). Cutting edge: cationic antimicrobial peptides block the binding of lipopolysaccharide (LPS) to LPS binding protein. J. Immunol. 164, 549–553. 10.4049/jimmunol.164.2.549 10623792

[B50] Sigurdardottir T. Andersson P. Davoudi M. Malmsten M. Schmidtchen A. Bodelsson M. (2006). *In silico* identification and biological evaluation of antimicrobial peptides based on human cathelicidin LL-37. Antimicrob. Agents Chemother. 50, 2983–2989. 10.1128/AAC.01583-05 16940092 PMC1563516

[B51] Spellberg B. Lipsky B. A. (2012). Systemic antibiotic therapy for chronic osteomyelitis in adults. Clin. Infect. Dis. 54, 393–407. 10.1093/cid/cir842 22157324 PMC3491855

[B52] Tang T. T. Lu H. Dai K. R. (2002). Osteogenesis of freeze-dried cancellous bone allograft loaded with autologous marrow-derived mesenchymal cells. Mater. Sci. Eng. C 20, 57–61. 10.1016/S0928-4931(02)00013-9

[B53] Tekin Koruk S. Aksoy N. Hamidanoglu M. Karsen H. Unlu S. Bilinc H. (2012). The activity of paraoxonase and arylesterase in patients with osteomyelitis. Scand. J. Clin. Lab. Invest. 72, 513–517. 10.3109/00365513.2012.700058 22950624

[B54] Tjabringa G. S. Aarbiou J. Ninaber D. K. Drijfhout J. W. Sorensen O. E. Borregaard N. (2003). The antimicrobial peptide LL-37 activates innate immunity at the airway epithelial surface by transactivation of the epidermal growth factor receptor. J. Immunol. 171, 6690–6696. 10.4049/jimmunol.171.12.6690 14662872

[B55] Wagner C. Hansch G. M. (2015). Pathophysiology of implant-associated infections: from biofilm to osteolysis and septic loosening. Orthopade 44, 967–973. 10.1007/s00132-015-3183-z 26556489

[B56] Wang G. (2008). Structures of human host defense cathelicidin LL-37 and its smallest antimicrobial peptide KR-12 in lipid micelles. J. Biol. Chem. 283, 32637–32643. 10.1074/jbc.M805533200 18818205

[B57] Wang Y. Ma J. Du Y. Miao J. Chen N. (2016). Human amnion-derived mesenchymal stem cells protect human bone marrow mesenchymal stem cells against oxidative stress-mediated dysfunction *via* ERK1/2 MAPK signaling. Mol. Cells 39, 186–194. 10.14348/molcells.2016.2159 26743906 PMC4794600

[B58] Wang Y. Wu H. Shen M. Ding S. Miao J. Chen N. (2017). Role of human amnion-derived mesenchymal stem cells in promoting osteogenic differentiation by influencing p38 MAPK signaling in lipopolysaccharide-induced human bone marrow mesenchymal stem cells. Exp. Cell Res. 350, 41–49. 10.1016/j.yexcr.2016.11.003 27832946

[B59] Wu H. Hu B. Zhou X. Zhou C. Meng J. Yang Y. (2018). Artemether attenuates LPS-induced inflammatory bone loss by inhibiting osteoclastogenesis and bone resorption *via* suppression of MAPK signaling pathway. Cell Death Dis. 9, 498. 10.1038/s41419-018-0540-y 29703893 PMC5924411

[B60] Xiao G. Gopalakrishnan R. Jiang D. Reith E. Benson M. D. Franceschi R. T. (2002). Bone morphogenetic proteins, extracellular matrix, and mitogen-activated protein kinase signaling pathways are required for osteoblast-specific gene expression and differentiation in MC3T3-E1 cells. J. Bone Miner. Res. 17, 101–110. 10.1359/jbmr.2002.17.1.101 11771655

[B61] Xing Q. Ye Q. Fan M. Zhou Y. Xu Q. Sandham A. (2010). Porphyromonas gingivalis lipopolysaccharide inhibits the osteoblastic differentiation of preosteoblasts by activating Notch1 signaling. J. Cell Physiol. 225, 106–114. 10.1002/jcp.22201 20648628

[B62] Yamazaki M. Fukushima H. Shin M. Katagiri T. Doi T. Takahashi T. (2009). Tumor necrosis factor alpha represses bone morphogenetic protein (BMP) signaling by interfering with the DNA binding of Smads through the activation of NF-kappaB. J. Biol. Chem. 284, 35987–35995. 10.1074/jbc.M109.070540 19854828 PMC2791026

[B63] Yeom M. Kim J. H. Min J. H. Hwang M. K. Jung H. S. Sohn Y. (2015). Xanthii fructus inhibits inflammatory responses in LPS-stimulated RAW 264.7 macrophages through suppressing NF-kappaB and JNK/p38 MAPK. J. Ethnopharmacol. 176, 394–401. 10.1016/j.jep.2015.11.020 26560439

[B64] Zasloff M. (2002). Antimicrobial peptides of multicellular organisms. Nature 415, 389–395. 10.1038/415389a 11807545

[B65] Zeng X. Tian J. Cai K. Wu X. Wang Y. Zheng Y. (2014). Promoting osteoblast differentiation by the flavanes from Huangshan Maofeng tea is linked to a reduction of oxidative stress. Phytomedicine 21, 217–224. 10.1016/j.phymed.2013.08.026 24075209

[B66] Zhang W. Liu H. T. (2002). MAPK signal pathways in the regulation of cell proliferation in mammalian cells. Cell Res. 12, 9–18. 10.1038/sj.cr.7290105 11942415

