# Supplementary figures and images for: KR-12-a5 Reverses Adverse Effects of Lipopolysaccharides on HBMSC Osteogenic Differentiation by Influencing BMP/Smad and P38 MAPK Signaling Pathways
Source: Front Pharmacol. 2019 Jun 5;10:639. doi: 10.3389/fphar.2019.00639 (PMC6561377; doi:10.3389/fphar.2019.00639)

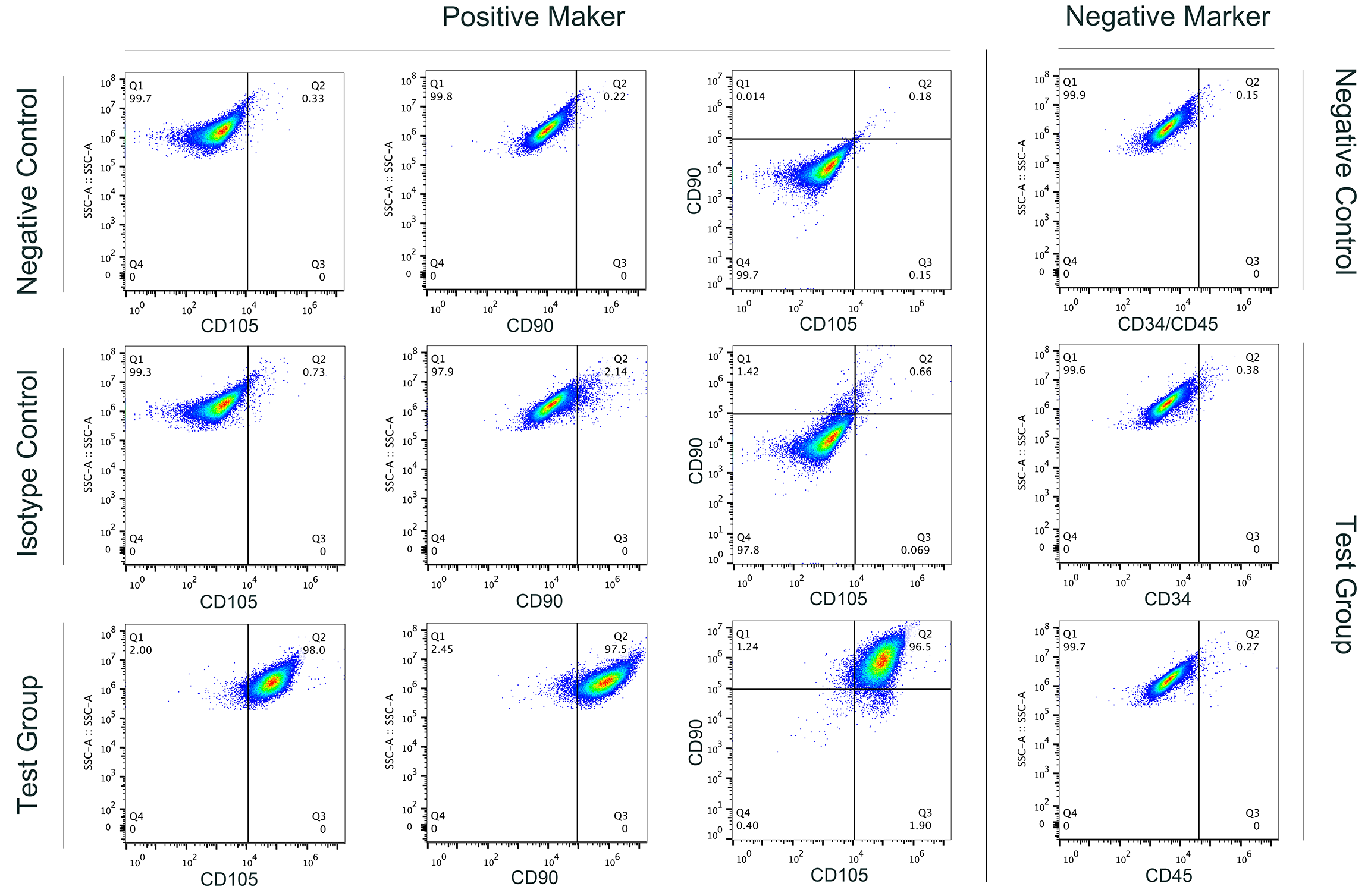

Supplement: Figure S1 — Surface marker analysis of HBMSCs by flow cytometry. [file Image_1.tif]
